# Myricetin alleviates testosterone-induced benign prostatic hyperplasia by attenuating inflammation, oxidative stress, apoptosis and androgen signaling

**DOI:** 10.1038/s41598-026-47374-0

**Published:** 2026-04-07

**Authors:** Ghada Alomari, Bahaa Al-Trad, Janti Qar, Lena Tahat, Yazan Abu Haija, Aseel Al-najjar, Ibrahim Albokhadaim, Saad Shousha, Ahmed O. Alameen, Mustafa Shukry

**Affiliations:** 1 Department of Biological Sciences, Faculty of Science, Yarmouk University, Irbid, 211-63 Jordan; 2 Department of Biomedical Sciences, College of Veterinary Medicine, King Faisal University, Al-Ahsa, 31982 Saudi Arabia

**Keywords:** Myricetin, Benign prostatic hyperplasia, DHT, 5-α reductase, Androgen receptors, Inflammation, Biochemistry, Diseases, Drug discovery, Medical research, Urology

## Abstract

This study investigates the effect of myricetin as a therapeutic for benign prostatic hyperplasia (BPH) disease using a testosterone-induced BPH animal model. Forty adult male rats were randomly divided into four groups as follows: control group, BPH group that was injected with testosterone subcutaneously (3 mg/kg body weight/day), BPH + myricetin group, which received myricetin (50 mg/kg) subcutaneously every day with the BPH induction, and BPH + finasteride group, which administered finasteride orally with the BPH induction at a daily dose of 5 mg/kg. After 28 days, blood and prostate tissue samples were collected for analysis. Compared to the control group, treatment with myricetin improved the pathohistological signs of BPH and enhanced the antioxidant and anti-inflammatory capacity of the prostatic tissue, as evidenced by its ability to enhance the total antioxidant capacity (TAC) levels and reduce malondialdehyde (MDA) levels, decrease the levels of the inflammatory biomarkers tumor necrosis factor alpha (TNF-α) and interleukin-1 beta (IL1-β) levels, and reduce serum dihydrotestosterone (DHT) levels. In addition, myricetin treatment showed beneficial effects through its ability to reduce the prostatic mRNA expression levels of the anti-apoptotic protein Bcl2, the *5-α reductase* enzyme, and the androgen receptor (*AR*), while simultaneously increasing the prostatic mRNA expression levels of the pro-apoptotic protein *Bax*. Myricetin treatment also exhibited anti-proliferative and anti-angiogenic effects, as evidenced by the reduced prostatic proliferating cell nuclear antigen (PCNA) and vascular endothelial growth factor-A (*VEGF-A*) mRNA expression levels. In summary, myricetin displays potential as a BPH therapy by diminishing inflammation and oxidative stress, hindering *5-α reductase*/*AR*/DHT signaling, and endorsing pro-apoptotic over anti-apoptotic pathways.

**Supplementary Information:**

The online version contains supplementary material available at 10.1038/s41598-026-47374-0.

## Introduction

Flavonoids are a large group of natural substance that shared chemical structure (C6-C3-C6 rings)^1^. Plants make flavonoids, which are basically pigment compounds, to protect themselves from dangerous insects and active oxygen that comes from being exposed to UV light. Flavonoids have recently garnered significant attention due to their diverse pharmacological properties, encompassing anti-oxidant, anti-inflammatory, anti-tumor, anti-diabetic, anti-obesity, anti-hypertensive, and anti-viral actions^1,2^. Nonetheless, the physiological impacts of flavonoids starkly contrast with their limited bioavailability, and the fundamental mechanism driving their protective effects on metabolism remains unclear^2^.

A valuable natural active flavonol component, myricetin (3,3,4,5,5,7-hexahydroxyflavone) is found in a wide variety of natural plants, including berries, vegetables, and herbs^3^. Myricetin is commonly used as a dietary supplement and has been demonstrated to have a variety of biological activities, including hepatoprotective, anti-inflammatory, antimicrobial, anticancer, antidiabetic, anti-obesity, osteoporosis prevention, and antioxidant properties^4–7^.

Benign prostatic hyperplasia (BPH) is one of the most prevalent urinary conditions affecting approximately 50–75% of men aged 50 years and older and increasing to around 80% by the age of 70. BPH considered a public global health issue due to its economic influence and its significant burden which impact quality of life^8^. Although onset of BPH primarily linked to androgenic imbalances, oxidative stress and chronic inflammation are additional factors that implicated in the pathophysiology of BPH^9^.

This multifactorial disease is more common as people age. Aging causes a decrease in the testosterone/estrogen ratio and a rise in estrogen activity, which may promote prostate cell hyperplasia. Another proposed mechanism involves the activity of the enzyme 5α-reductase, which converts testosterone to the more potent dihydrotestosterone (DHT). This enzyme’s activity rises with age in men, resulting in a lower testosterone/DHT ratio. Prostate cell growth may be stimulated by DHT, leading to hyperplasia. Therefore, some therapeutic plants and their compounds have targets this enzyme^10,11^.

Consequently, the utilization of 5α-reductase inhibitors, including finasteride, constitutes a logical therapeutic strategy for conditions associated with 5α-reductase activity, notably BPH. These agents lower DHT levels, which leads to a reduction in prostate volume, improvement in maximum urinary flow rates, and relief of symptoms associated with the static mechanical obstruction characteristic of BPH^12^. But these therapies do not directly modulate oxidative stress or inflammatory cascades^9^. In addition, these treatments are not entirely effective due to the potential for unwanted effects, including weakness, tiredness, trouble sleeping, sexual dysfunction, and prostate cancer^13^.

Natural products could serve as novel, effective, and safe medicinal treatment if adequately evaluated^10^. This study evaluated the treatment efficacy of myricetin against BPH and investigated its mechanism of action. The rationale for investigating myricetin in the treatment of BPH is strongly supported by its pro-apoptotic, anti-proliferative, and anti-inflammatory activities, initially evidenced in prostate cancer cells^14,15^, which are directly applicable to the pathogenesis of BPH.

## Materials and methods

All experimental protocols were reviewed and approved by animal ethics committee at Yarmouk University under approval number (IACUC/2024/1). All procedures were carried out in strict accordance with the relevant institutional, national, and international guidelines and regulations governing the care and use of laboratory animals. Furthermore, all methods are reported in accordance with the ARRIVE guidelines (https://arriveguidelines.org/) to ensure transparency and reproducibility in animal research.

Adult male Wistar rats (n = 40, weighing (284.6 g ± 19.66) were obtained from the animal facility at Yarmouk University (Irbid, Jordan). Sample size was determined according to resource equation method^16^. The animals were maintained in a 12-h light and 12-h dark cycle, with regulated temperature (22–25 °C) and relative humidity (30–40%). The rats were provided with water and fed ad libitum with a standard laboratory rodent chow. Animals were given one week to adjust to the lab environment before beginning the study.

The rats were randomly assigned to four groups of ten each, as follows: Group 1 is the control group (without BPH) that received 200 µl corn oil as a vehicle administered subcutaneously once daily; in groups 2, 3, and 4, BPH was induced in rats by administering testosterone Sustanon 250 (Durban, KZN, and SA) subcutaneously (3 mg/kg body weight/day, dissolved in corn oil)^17^; additionally, group 3 is the (BPH + Myricetin) group, which received Myricetin (50 mg/kg dissolved in minimum volume of DMSO (5% DMSO, 95% Normal saline)) subcutaneously every day with the BPH induction, and last group (finasteride, Fin) received finasteride (dissolved in normal saline) orally by gastric gavage at a daily dose of 5 mg/kg. All treatments were administered in a standardized volume of 200 µl per animal to ensure consistency across experimental groups. The selected myricetin dose was based on previous in vivo rodent studies demonstrating the dose 50 mg/kg effectively exert anti-inflammatory, antioxidant, and antiproliferative effects without inducing systemic toxicity^18,19^. Myricetin was sourced from Biosynth, CAS (529-44-2), and finasteride sourced from ADWIA, Egypt. The treatment duration was 28 days. One day following the last administration, the rats were weighed and euthanized with sodium pentobarbital (100 mg/kg; i.p.), followed by the collection of blood and ventral lobes of prostate tissue. Harvested prostate tissues were weighed then divided into two parts: one part immersed in 10% neutral buffered formalin for histological investigation, the other part immediately snap-frozen in liquid nitrogen and stored at − 80 °C until protein analysis and RNA extraction. For protein analysis 0.2 g of tissue from each prostate were homogenized in 1000 μL of 1 × PBS, followed by centrifugation at 11,200×*g* using cooling centrifuge (HERMLE, Z 326 K, Germany) for 10 min at 4 °C. The supernatants were collected and stored at − 80 °C for subsequent analyses.

### Histopathological examination

Prostatic tissues were dissected out, washed with normal saline, and fixed in 10% phosphate-buffered formalin solution at room temperature overnight. After that, specimens were dehydrated in a graded series of ethanol, cleared in xylene, and embedded in paraffin. Sections of prostate (4–5 µ in thickness) were obtained, mounted on slides, and kept at room temperature. Later, slides were stained with hematoxylin and eosin and evaluated under light microscopy. 15 randomly selected prostatic acini per animal were analyzed to measure epithelial thickness. Histopathological alterations and histomorphometric measurements were assessed by an investigator blinded to the group treatments.

### Assessment of prostatic levels of the proliferative biomarker PCNA, the inflammatory markers TNF-α and IL-1β

To elucidate the impact of myricetin on the mechanisms underlying BPH development, the protein levels of the inflammatory markers TNF-α and IL-1β, along with the proliferative biomarker PCNA, in prostate tissue were assessed utilizing commercial ELISA kits (Wuhan Fine Biotech, China) according to the manufacturer’s guidelines. TNF-α (Catalog No. ER1393, sensitivity 2.344 pg/mL, coefficients of variation < 10%), IL-1β (Catalog No. ER1094, sensitivity 18.75 pg/mL, coefficients of variation < 10%), and PCNA (Catalog No. R1234, sensitivity ~ 0.094 ng/mL, coefficients of variation < 10%), Absorbance was measured using Multiskan SkyHigh Microplate Spectrophotometer A51119500C (Thermo Scientific, USA), and concentrations were calculated from standard curves generated for each assay.

### Determination of lipid peroxidation biomarker (MDA) and total antioxidant capacity (TAC) levels

The prostate MDA level was assesses utilizing the subsequent methodology: 15% trichloroacetic acid (TCA), 0.375% (w/v) thiobarbituric acid (TBA), and 0.25 M hydrochloric acid (HCl) were combined to create a TCA-TBA-HCl reagent, which was then heated. Tissue samples were diluted 1:100 in PBS to make a total volume of 1 mL. Each diluted sample was then mixed with 2 mL of the TCA-TBA-HCl reagent, heated at 95 °C for 30 min, cooled, and the absorbance measured at 535 nm. The MDA levels were calculated using a molar extinction coefficient of 1.56 × 10^5^ M⁻^1^ cm⁻^1^ and expressed as nmol MDA per mg tissue^20^. TAC in prostate tissue was measured using a TAC activity assay kit (REF. No. 100T-96S Genochem World, Spain), according to the manufacturer’s instructions.

### Analysis of serum dihydrotestosterone (DHT) levels

Given that DHT is pivotal in the pathogenesis of BPH, serum DHT levels were quantified utilizing a commercial ELISA kit DHT (Catalog No. EA0046Ra, BT LAB, Zhejiang, China) following the manufacturer’s guidelines.

### Gene expression by reverse transcription quantitative real-time PCR (RT-qPCR)

Prostate tissues were collected at the end of the experiment for examining the expression levels of genes encoding *Bax*, *Bcl-2*, vascular endothelial factor-A (*VEGF-A*), *5-α reductase*, and androgen receptor (*AR*). The isolation of total RNA was performed utilizing the GeneJET RNA Purification Kit (Thermo Scientific™, USA), reverse-transcribed into complementary DNA (cDNA) using the EasyScript® First-Strand cDNA Synthesis Super-Mix (TransGen Biotech, China). Quantitative real-time PCR (qRT-PCR) for the genes’ mRNA levels was performed using the Line-Gene 9600 Real-Time PCR system (Bioer Technology, Bingjiang, China). The transcript levels were standardized using glyceraldehyde 3-phosphate dehydrogenase (*GAPDH*) as an endogenous control. All gene-distinct primer sequences used in the RT-qPCR assay are previously listed in a previous work^17^ and provided in supplementary Table 1. The relative mRNA expression of the studied genes was evaluated using the 2^−ΔΔCt^ method^21^. Primer efficiency was validated using standard curve analysis and was found to be close to 100% for all primer pairs, supporting the use of the 2^−ΔΔCt^ method for relative quantification. Glyceraldehyde-3-phosphate dehydrogenase (*GAPDH*) was used as the reference gene, and its expression did not differ significantly among experimental groups, as confirmed by one-way ANOVA (*P* > 0.05), indicating stable expression under the experimental conditions.

### Statistical analysis

Statistical analyses of the data were performed with Graph Pad Prism 7.01 (Graph Pad Software Inc., CA, USA; https://www.graphpad.com/scientific-software/prism/) Data are expressed as the mean ± standard error of the mean (SEM). To compare differences between groups, one-way ANOVA followed by Tukey’s multiple comparisons was performed. Significant differences were considered when *P* < 0.05.

## Results

### Myricetin prevented enlargement of prostate and morphological changes in BPH rat model

**Fig. 1:**
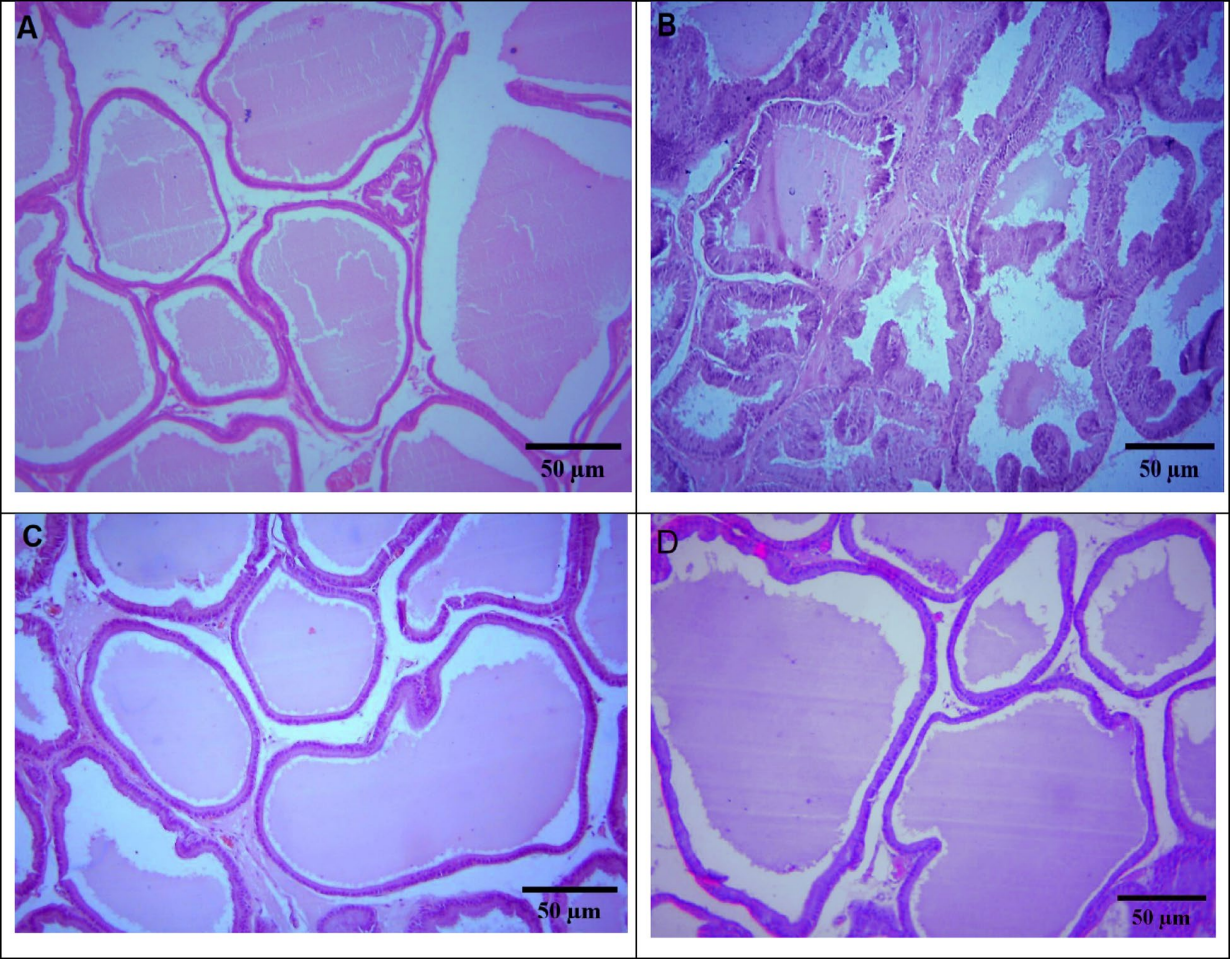
Histological analysis of prostate tissues. Control group shows normal architecture (**A**). benign prostatic hyperplasia (BPH) group displays epithelial proliferation and papillary fold formation (**B**). Myricetin (**C**) and Finasteride (**D**) treatments markedly reduced epithelial proliferation compared with the BPH group.

**Fig. 2:**
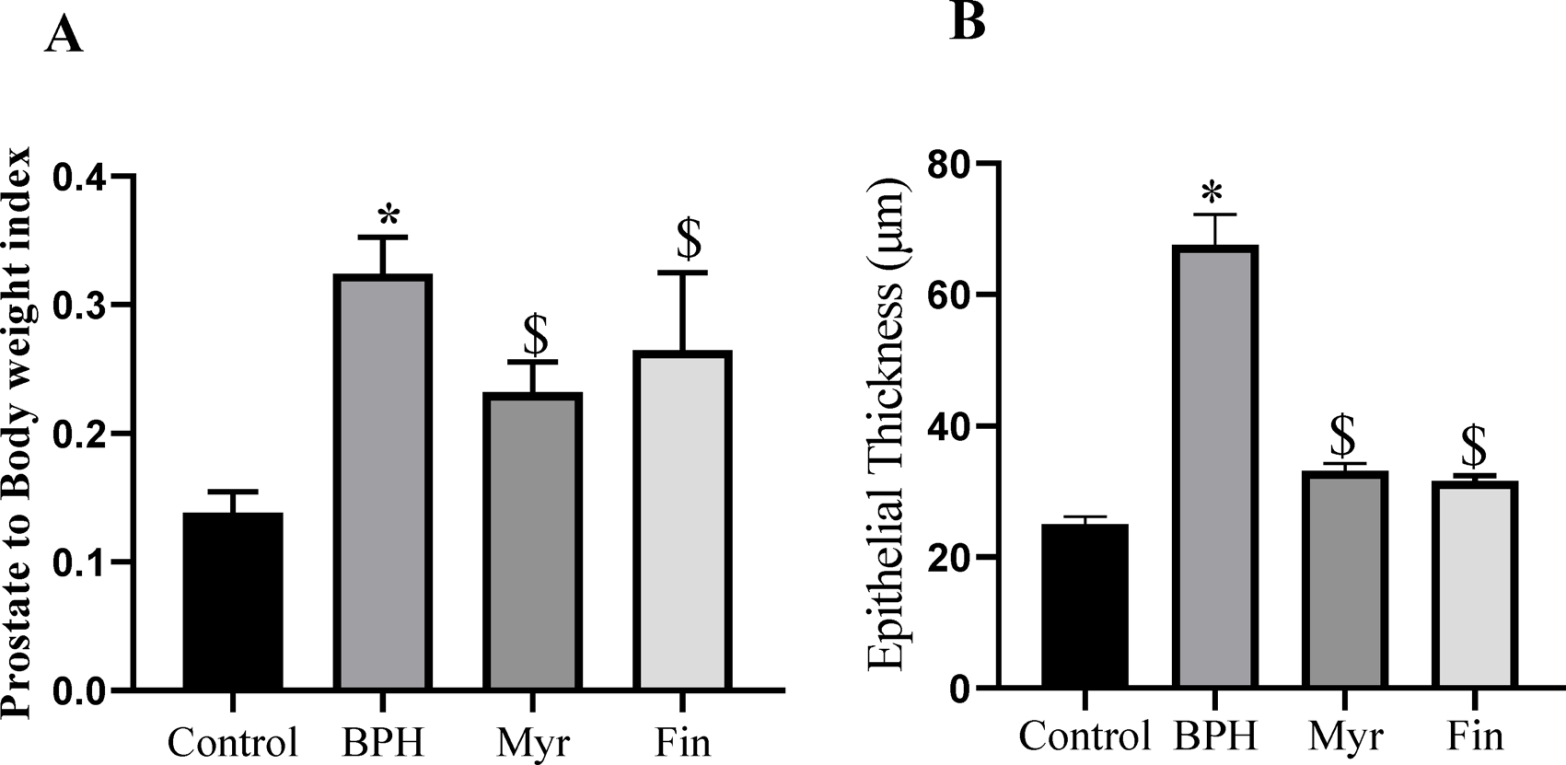
Quantitative analysis. (**A**) Prostate-to-body weight index and (**B**) epithelial thickness measurements, both consistent with the histological observations. Sample size n = 8–10. Data represent mean ± SEM. Using one-way ANOVA *P < 0.05 compared to the control group. $ *P* < 0.05 compared to benign prostatic hyperplasia (BPH). Myr: Myricetin, Fin: Finasteride.

Histological evaluation of prostate tissues is shown in Fig. 1. In the BPH group, the development of prostatic hyperplasia was evident when compared to the intact control group, characterized by morphological alterations such as epithelial proliferation within the glandular regions and the formation of papillary folds (Fig. 1B). Treatment with myricetin and finasteride markedly reduced the proliferative activity of the prostatic epithelium relative to the BPH group (Fig. 1C,D). These findings were further supported by the prostate-to-body weight index (Fig. 2A) and measurements of prostate epithelial thickness (Fig. 2B), which were consistent with the histological observations.

### Antioxidant properties

**Fig. 3:**
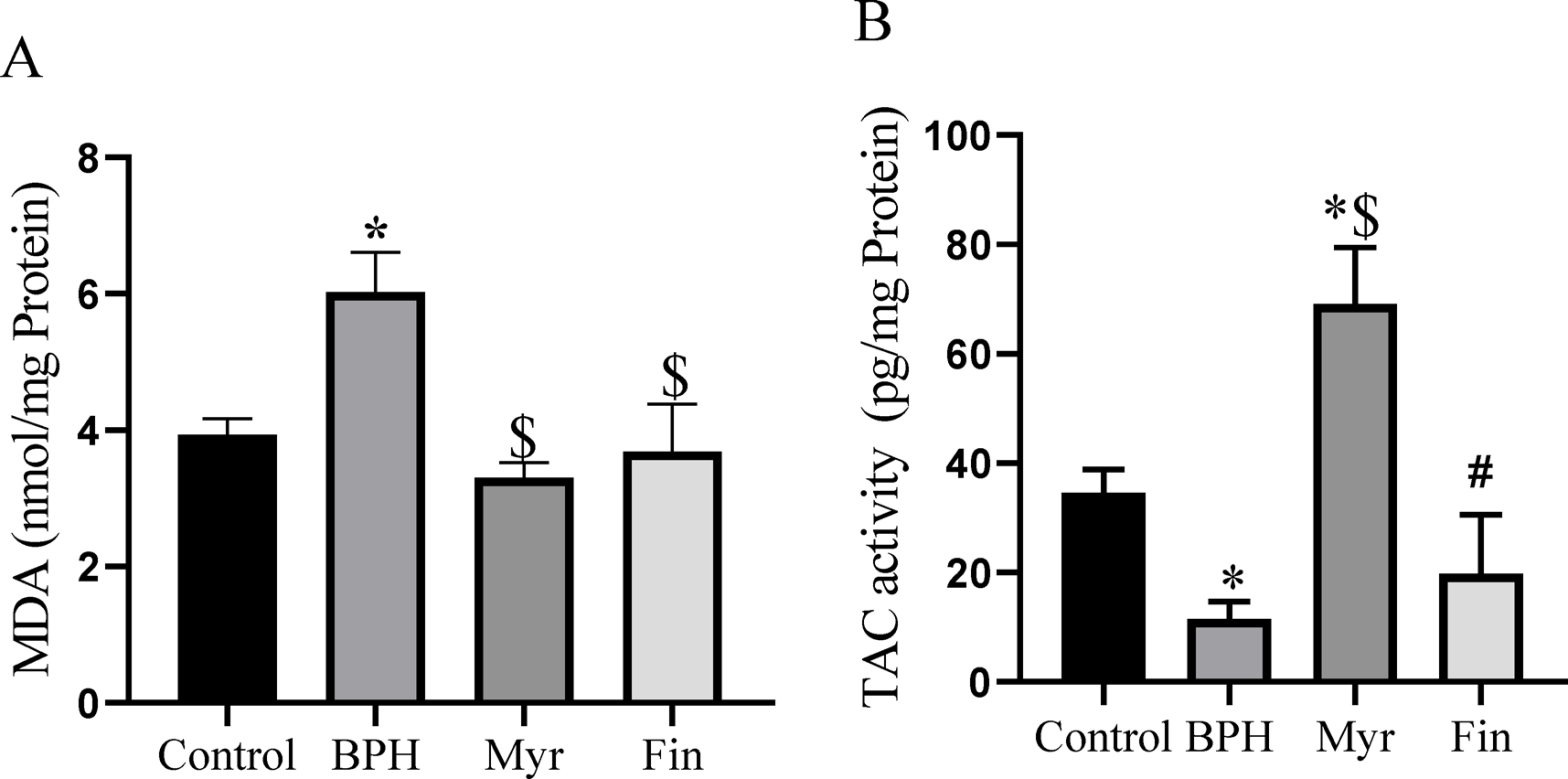
(**A**) MDA concentration in prostate tissue (nmol/mg protein) reduced significantly after myricetin (Myr) treatment. (**B**) TAC significantly upregulated in Myr group compared to all other groups. Sample size n = 8–10. Data represent mean ± SEM. Using one-way ANOVA *P < 0.05 compared to the control group. $ *P* < 0.05 compared to benign prostatic hyperplasia (BPH). # *P* < 0.05 compared to Myr. Fin: Finasteride.

Oxidative stress is one of the parameters that plays a key role in the development of BPH. To evaluate the anti-oxidative effects of myricetin in the prostate, the lipid peroxidation marker malondialdehyde (MDA) and TAC levels were measured. Animals with BPH exhibited a significant elevation in MDA levels, confirming the induction of oxidative stress in this group (*P* < 0.05). In contrast and as shown in Fig. 3A, treatment with either myricetin or finasteride significantly reduced MDA levels compared with the BPH group (*P* < 0.05). No significant differences in MDA concentration were detected between the myricetin, finasteride, and control groups. Analysis of TAC levels revealed that myricetin group exhibited a significant elevation compared with all other groups. In contrast finasteride did not result in a significant change in TAC levels relative to the other groups (Fig. 3B).

### The effects of myricetin on prostatic IL-1β and TNF-α levels

**Fig. 4:**
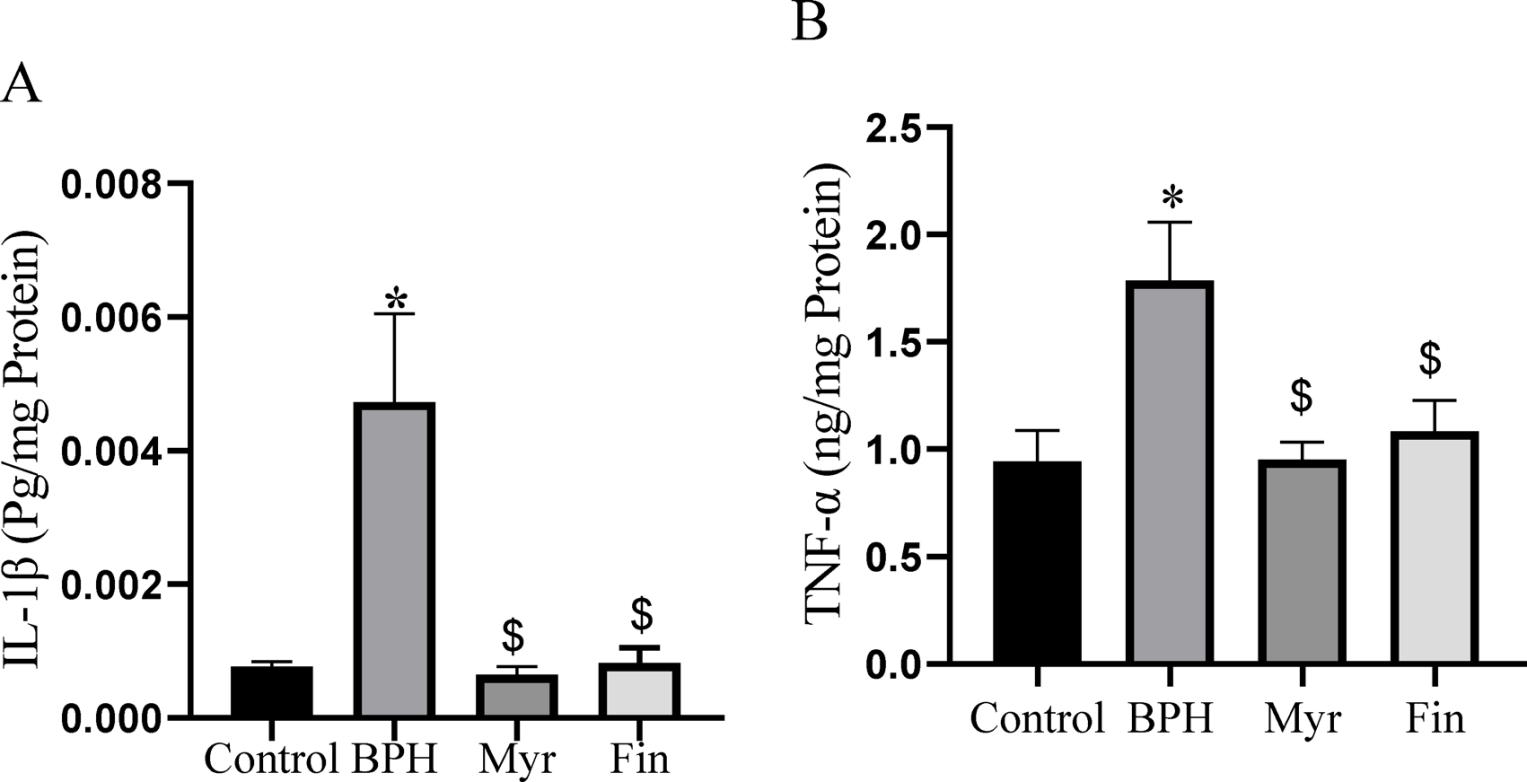
IL-1β concentration (ng/mg protein) (A), and TNF-α concentration (pg/mg protein) (B) in prostatic tissue downregulated after myricetin (Myr) treatment. Sample size n = 8–10. Data represent mean ± SEM. Using one-way ANOVA *P < 0.05 compared to the control group. $ *P* < 0.05 compared to benign prostatic hyperplasia (BPH). Myr: Myricetin, Fin: Finasteride.

Inflammation is an important causative agent in the progress of BPH. In order to determine possible mechanisms of myricetin-mediated prostatic protection, the levels of the inflammatory cytokines IL-1β and TNF-α in prostatic tissue were measured. As shown in Fig. 4 A &B, compared to the control group, the levels of IL-1β and TNF-α increased significantly in the BPH group (*P* < 0.05). Furthermore, compared to the BPH group, the level of these pro-inflammatory cytokines were significantly decreased in the myricetin and finasteride treated groups (*P* < 0.05).

### Effects of myricetin on androgen signaling, serum dihydrotestosterone (DHT), and *5-α reductase* mRNA expression

**Fig. 5:**
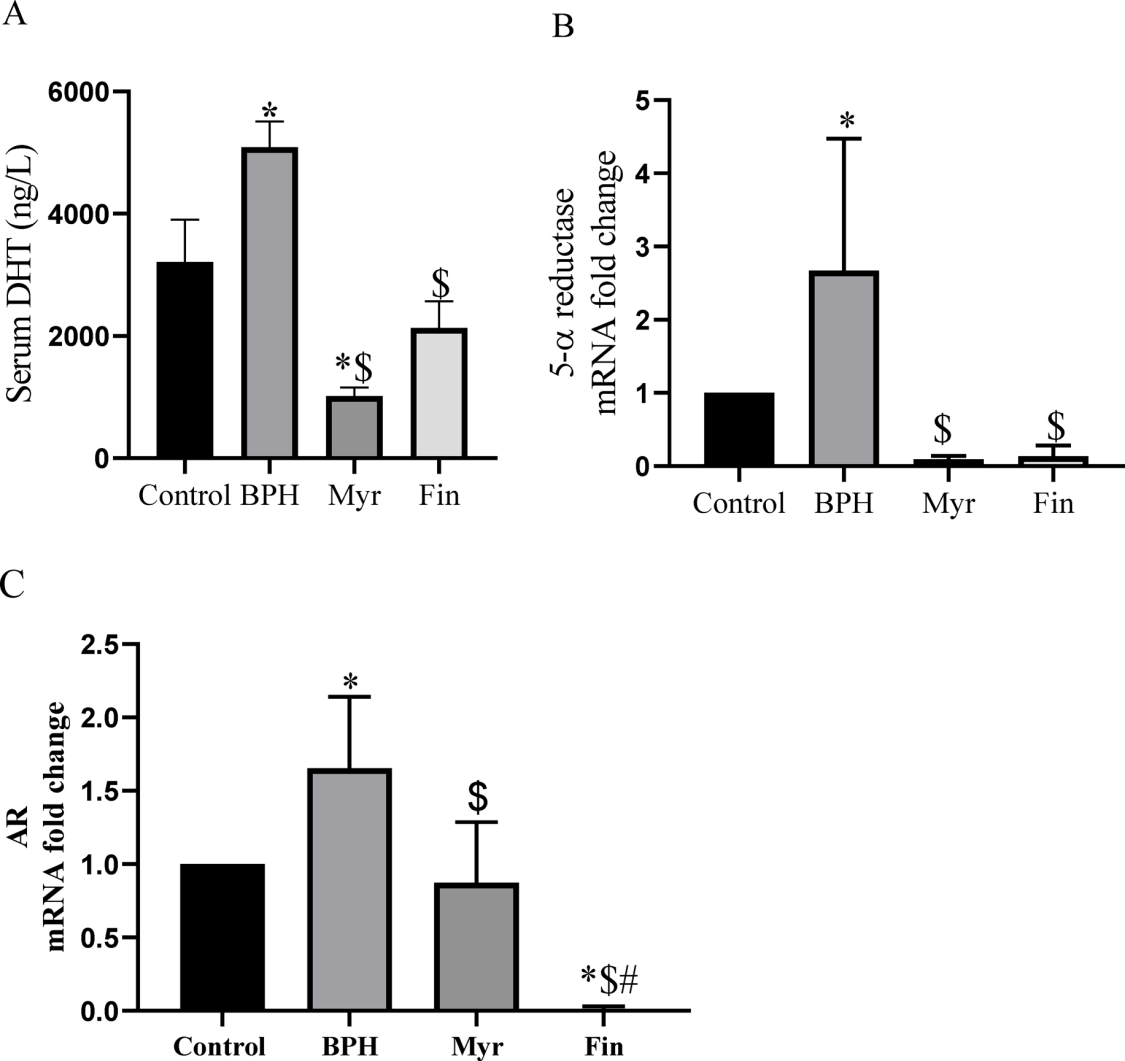
Serum Dihydrotestosterone (DHT) concentration (ng/ml) (**A**), *5α- reductase* mRNA expression in prostatic tissue (**B**), and androgen receptor (*AR*) mRNA expression in prostatic tissue (**C**), all reduced significantly after myricetin (Myr) treatment. Sample size n = 8–10. Data represent mean ± SEM. Using one-way ANOVA *P < 0.05 compared to the control group. $*P* < 0.05 compared to benign prostatic hyperplasia (BPH). #*P* < 0.05 compared to Myr groups. Myr: Myricetin, Fin: Finasteride.

Activation of the nuclear androgen receptors (*AR*) by DHT, a potent prostatic androgen that produced by reduction of testosterone in presence of 5-α reductase enzyme, all plays a pivotal role in the pathogenesis of prostate hyperplasia. BPH group was accompanied by a significant increase in serum DHT levels (*P* < 0.05 vs. intact control) as shown in Fig. 5 A. Treatment with myricetin significantly decreased DHT levels compared with both the control and the BPH groups (*P* < 0.05), whereas finasteride produced a significant reduction only when compared with the BPH group (*P* < 0.05). These findings are simultaneous with the gene expression results, showing that *5-α reductase* and *AR* mRNA expression were significantly downregulated in myricetin treated group, whereas both were significantly upregulated in the BPH group (*P* < 0.05), (Fig. 5B,C). In the finasteride-treated group, *5-α reductase* mRNA expression was significantly downregulated compared with the BPH group, while *AR* mRNA expression was significantly downregulated relative to all other groups; the control, BPH, and myricetin-treated groups.

### Myricetin pro-apoptotic, anti-angiogenic, and anti-proliferative actions

**Fig. 6:**
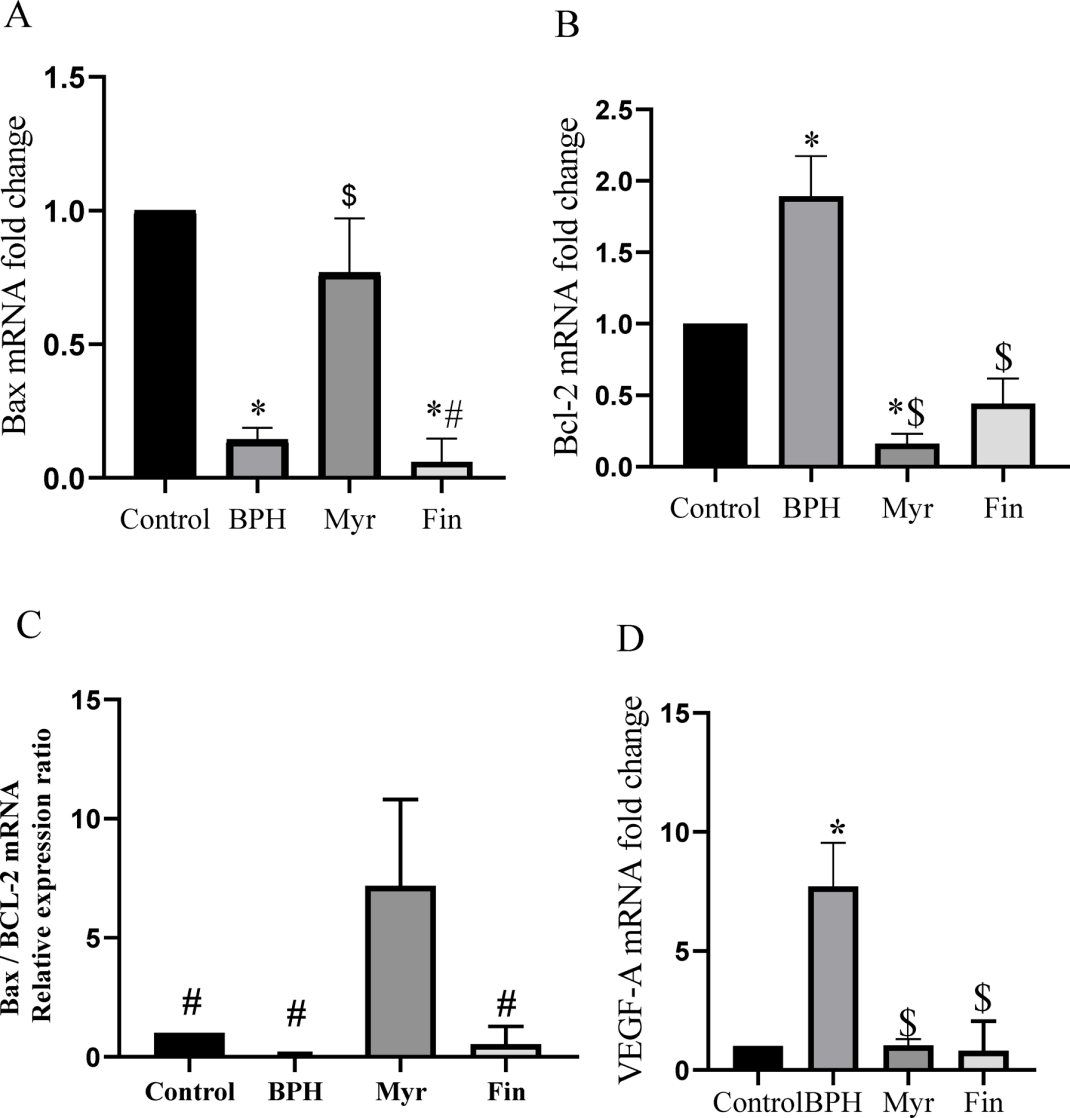

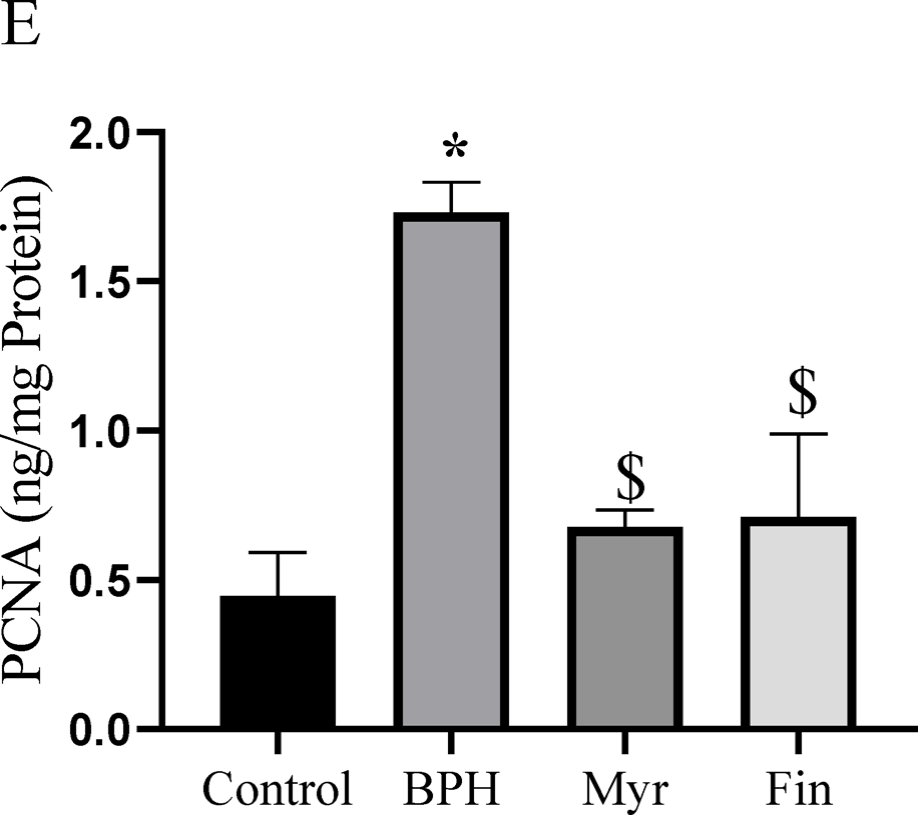
Myricetin (Myr) treatment upregulated the relative expression of *Bax* (**A**), while *Bcl-2* was downregulated relative to the BPH group (**B**). Bax/Bcl-2 ratio upregulated in Myr group relative to the BPH group (**C**). *VEGF-A* mRNA expression levels (**D**) and PCNA protein levels (**E**), were significantly decreased after myricetin treatment. Sample size n = 8–10. Data represent mean ± SEM. Using one-way ANOVA *P < 0.05 compared to the control group. $*P* < 0.05 compared to benign prostatic hyperplasia (BPH). #*P* < 0.05 compared to Myr group. Fin: Finasteride.

The imbalance between cellular proliferation and apoptosis results in abnormal enlargement of the prostate gland, leading to BPH. To investigate the effect of myricetin on apoptosis, as shown in Fig. 6A,B, the mRNA expression of *Bax* and *Bcl-2* was evaluated. In the BPH group, the proapoptotic factor *Bax* was significantly downregulated (*P* < 0.05), and the anti-apoptotic factor *Bcl-2* was significantly elevated compared to the control group (*P* < 0.05). Treatment of myricetin upregulated the expression of *Bax*, whereas the expression of *Bcl-2* was downregulated relative to the control and BPH groups (*P* < 0.05). In the finasteride-treated group, *Bax* mRNA expression did not differ from the BPH group but was significantly lower compared to myricetin and control groups (*P* < 0.05), whereas *Bcl-2* expression was significantly downregulated compared with BPH group (*P* < 0.05). Myricetin treatment significantly increased the Bax/Bcl-2 ratio compared to the other groups (*P* < 0.05) (Fig. 6C), indicating a shift toward a pro-apoptotic state. The mRNA expression of the angiogenic biomarker *VEGF-A* (Fig. 6D) exhibited a significant elevation in the BPH group relative to the control group (*P* < 0.05), nonetheless, it was markedly diminished in the myricetin and finasteride treated groups compared to BPH group (*P* < 0.05). As illustrated in Fig. 6E, the proliferative marker PCNA’s protein levels were significantly elevated in the BPH group compared with the controls. Myricetin and finasteride treatment markedly reduced PCNA levels relative to the BPH group (*P* < 0.05). These findings suggest that myricetin modulates angiogenesis, proliferation, and apoptosis during BPH progression in this animal model.

## Discussion

Prostate gland weight, epithelial proliferation, oxidative stress, and blood serum DHT levels all increased in tandem with the onset of BPH. At a dose of 50 mg/kg, myricetin significantly inhibited the development of BPH, as seen by a drop in prostate weight and epithelial proliferation, the decrease in blood serum DHT, a decrease in oxidative stress measured by MDA, and a decrease in *AR* and *5-α reductase* mRNA expression levels.

Inflammation plays a crucial role in the onset and development of BPH, with prostatic inflammation arising from overlapping factors including infection, immunological responses, hormonal imbalance, and urinary reflux^22^. Proinflammatory cytokines like TNF-α and IL-6, have been shown to promote nodular hyperplasia by driving cellular proliferation and blocking normal apoptotic turnover^9^. In addition, under inflammatory conditions, prostate epithelial cells can undergo epithelial-mesenchymal transition and fibrotic remodeling of prostate, which linked to disease progression^23,24^. Accordingly, inflammation could be a potential therapeutic target of managing BPH^25^.

Several studies have demonstrated that myricetin exhibits unique anti-inflammatory properties across a range of signal modulations in several inflammatory disease models^5^. In myocardial and hepatic injury models, myricetin treatment was associated with reduced tissue damage and decrease levels of proinflammatory cytokines, including TNF-α, IL-1β, and IL-6^26,27^. These reports support the present findings, in which myricetin treatment was associated with reduced the IL-1β and TNF-α levels in prostate tissue, potentially contributing to the relief of BPH-related histopathological alterations.

Growing evidence indicates that, in addition to chronic inflammation, oxidative stress plays a pivotal role in the pathogenesis of BPH^5,9^. Excessive reactive oxygen species, produced either during normal metabolic activity or in response to enhanced inflammatory processes, can surpass the capacity of endogenous antioxidant systems. This imbalance contributes to fibromuscular proliferation and impaired regulation of apoptosis, ultimately leading to lower urinary tract symptoms (LUTS) such as increased urinary frequency, nocturia, and obstructive voiding difficulties^9^. Previous studies have demonstrated that agents with antioxidant properties exert protective effects against BPH, suggesting that preservation of the prostate’s antioxidant capacity may help prevent disease progression^9,28^.

Myricetin is recognized for its strong antioxidant properties, which play a key role in mitigating oxidative stress^29^. These effects are largely attributed to its high polarity and ability to permeate cell membranes, enabling efficient suppression of intracellular reactive oxygen species (ROS)^30^. The potent antioxidant capacity of myricetin, compared with other flavonoids, is primarily due to the presence of three hydroxyl groups on its B-ring, which facilitate interactions with multiple enzymes and receptors^4,31^. Moreover, myricetin exerts its antioxidative effects by upregulating the activity of endogenous antioxidant enzymes in experimental rodent models, including superoxide dismutase (SOD), glutathione peroxidase (GPx), catalase (CAT), and glutathione (GSH), thereby attenuating inflammatory responses^32,33^. Consequently, myricetin has been proposed as a potential therapeutic agent for diseases associated with oxidative stress^34^.

In this study, malondialdehyde (MDA) and total antioxidant capacity (TAC) were evaluated to determine the protective effect of myricetin, a potent antioxidant, in prostatic tissue under BPH conditions. Myricetin administration significantly decreased MDA levels while enhancing TAC, confirming its antioxidant activity. These findings align with previous reports highlighting the efficacy of myricetin in alleviating oxidative stress. For instance, myricetin has demonstrated neuroprotective properties in both in vitro and in vivo models of Alzheimer’s disease by suppressing reactive oxygen species formation, lipid peroxidation, and DNA oxidation^35^. Similarly, another study reported that myricetin exerted hepatoprotective effects against acute liver failure by suppressing inflammation and modulating oxidative stress, as evidenced by reduced MDA levels and enhanced SOD and CAT activities^36^. Moreover, in an in vivo model of chronic obstructive pulmonary disease, myricetin treatment significantly decreased MDA content and increased SOD activity, further supporting its antioxidative potential^37^.

Prostate development, whether physiological or pathological, is primarily regulated by dihydrotestosterone (DHT), a testosterone metabolite with greater binding affinity for the androgen receptor. The conversion of testosterone to DHT is mediated by the enzyme 5-α-reductase. Pharmacological inhibition of this enzyme reduces both systemic and intraprostatic DHT levels, leading to regression of prostatic tissue and attenuation of BPH progression^38^. Pharmacological 5α-reductase inhibitors reduce AR signaling, thereby decreasing prostate volume and alleviating symptoms in a subset of BPH patients^39^.

In the present study, the suppressive effect of myricetin on BPH development appears to be associated with reduced DHT levels and downregulation of the mRNA expression of *5α-reductase* and *AR*, suggesting a potential modulatory effect on androgen signaling pathway. Previous studies have suggested that the activity of flavonols on the androgen AR pathway in prostate cancer is largely attributed to the presence of three hydroxyl groups on the B-ring. The proposed mechanisms include inhibition of 5α-reductase, direct competition with androgens for binding, interference with AR complex assembly, and attenuation of AR co-regulator-mediated transactivation^40^. However, future studies employing enzyme activity assays, protein-level analyses, and molecular docking approaches are warranted to clarify the direct molecular targets of myricetin in BPH.

In addition to reducing DHT levels, potentially through downregulation of the mRNA expression of *5α-reductase* and the *AR*, myricetin may also contribute to androgen balance through complementary mechanisms, including modulation of androgen synthesis and metabolism and antioxidant activity^41,42^. These mechanisms help explain the reduction in serum DHT levels which fall below control values as observed in the current study. Future studies evaluating serum testosterone levels and enzymatic pathways involved in androgen synthesis and metabolism will be important to fully understand the hormonal effects of myricetin in BPH.

Several factors contribute to the development of BPH, including disruption of the prostate’s normal balance between cell proliferation and apoptosis. Prostatic inflammation is a major driver, leading to both stromal and glandular hyperplasia. In addition, testosterone influences the expression of key regulatory genes in the prostate, such as *cyclin-D1*, *PCNA*, *Bax*, and *Bcl-2*, thereby promoting proliferative activity while inhibiting apoptotic pathways^11^.

PCNA, an essential regulator of DNA replication and cell cycle progression, plays a critical role in prostatic cell proliferation, and its absence has been associated with cell cycle arrest in prostatic tissue^43^. Therefore, PCNA is used as a marker protein of proliferating cells. In the present study, administration of myricetin resulted in a significant reduction of PCNA protein levels in the prostatic tissue. This reduction in PCNA levels suggests that myricetin effectively suppresses DNA replication and cell cycle progression, thus attenuating abnormal cellular proliferation. These findings indicate that the anti-proliferative action of myricetin may contribute to its protective role against BPH development, potentially through the restoration of the balance between proliferation and apoptosis in prostatic cells. These findings are consistent with previous reports demonstrating that myricetin suppresses the expression of cyclin D1 and PCNA in intestinal adenomatous polyps, consequently inhibiting malignant progression^44^. Similarly, in human breast cancer MCF-7 cells, myricetin markedly inhibited the PCNA signaling pathway and reduced cell viability^45^.

Apoptotic signaling is primarily governed by members of the *Bcl-2* protein family, where proteins such as *Bcl-2* and Bcl-XL act in an anti-apoptotic manner, while *Bax* and Bak function as pro-apoptotic factors. The balance between these opposing proteins determines the progression or inhibition of apoptosis^46^. The present study demonstrated that finasteride exhibited minimal modulation of apoptosis-related marker, *Bax*. This observation aligns with a previous report showing that finasteride reduced *Bax* expression, resulting in a decreased rate of apoptosis following the skin flap procedure^47^. In contrast, other study reported that finasteride can promote apoptosis in LNCaP cells via the involvement of the *Bcl-2* and caspase family proteins^48^. In comparison, treatment with myricetin was found to elevate the expression of the pro-apoptotic protein *Bax* while decreasing the expression of the anti-apoptotic protein *Bcl-2*. This regulatory shift indicates that myricetin promotes apoptosis in prostatic tissue through modulation of *Bax* and *Bcl-2* levels as reflected by the increased Bax/Bcl-2 ratio, consistent with the established concept that a reduced Bcl-2/Bax ratio promotes apoptotic processes^49^. These findings align with previous reports showing that myricetin exerts anticancer effects, such as inducing apoptosis via the MAPK pathway and regulating JNK-dependent autophagy in SK-BR-3 cells^46^. Similarly, studies in HepG2 cells have shown that myricetin facilitates apoptosis through Bax translocation to the mitochondria, suppression of *Bcl-2* expression, and upregulation of the pro-apoptotic protein Bad^50^.

Another crucial factor implicated in BPH is angiogenesis (construction of new blood vessels) in the prostatic gland. Under the influence of androgens, epithelial cells secrete the VEGF and fibroblast growth factor (bFGF), which endorse prostate cell proliferation leading to BPH^51^. Overexpression of VEGF and the epidermal growth factor receptor (EGFR) has been associated with increased vascularization and cellular proliferation in prostate tissues, consequently, hindering growth factor signaling may help slow down the development of prostate tissue and treating the underlying causes of BPH^52^.

Numerous studies have demonstrated the antiangiogenic potentials of myricetin. It was found to prevent breast tumor growth and angiogenesis via regulating the VEGF-mediated signaling pathways^7^. In another study, myricetin downregulated *VEGF-A* expression and inhibit neovascularization and proliferation in developmental systems^53^. In line with these reports, the present findings suggest that myricetin-associated antiangiogenic action may contribute to the attenuation of prostatic hyperplasia observed in this study.

Finasteride, a widely used 5α-reductase inhibitor, is commonly approved for the management of BPH, where it lowers testosterone and DHT levels in both serum and prostatic tissue, leading to reduced prostate volume and improvement of lower urinary tract symptoms^54^. Despite its clinical effectiveness, long-term finasteride therapy is associated with notable adverse effects, including sexual dysfunction and gynecomastia, which has prompted ongoing efforts to identify safer alternative treatments^55^.

In context of the current study, the present findings suggest that myricetin may represent a promising natural alternative for BPH management. Myricetin treatment was associated with simultaneous reductions in inflammatory cytokines (TNF-α, IL-1β), decreased oxidative stress, reduced DHT levels, and downregulation of *5-α reductase* and *AR* mRNA expression. Additionally, modulation of apoptotic, angiogenic and proliferative markers. While these effects appear coordinated, causal mechanisms remain unclear. Future studies targeting specific pathways are needed to identify its primary molecular targets and clarify underlying mechanisms.

## Conclusion

This study highlights the therapeutic potential of myricetin. Its multifunctional properties were associated with the protective effects in BPH-induced rats, targeting inflammation, oxidative stress, mRNA expression of *5-α reductase* and *AR*, and DHT levels, along with modulation of apoptotic and proliferative markers. While promising, the primary molecular targets and mechanisms remain to be clarified. Future mechanistic studies and clinical trials are needed to confirm its efficacy and address translational challenges such as bioavailability, formulation development, and long-term safety.

## Supplementary Information

Below is the link to the electronic supplementary material.

Supplementary Material 1

## Data Availability

All data generated or analyzed during this study are included in this published article.
