# Supplementary material for: Myricetin alleviates testosterone-induced benign prostatic hyperplasia by attenuating inflammation, oxidative stress, apoptosis and androgen signaling
Source: Sci Rep. 2026 Apr 7;16:11651. doi: 10.1038/s41598-026-47374-0 (PMC13061914; doi:10.1038/s41598-026-47374-0)
Supplement: Supplementary file 1 — Supplementary Material 1 [file 41598_2026_47374_MOESM1_ESM.docx]

| Gene | Forward (5'-3') | Reverse (5'-3') | Accsesion Number | Amplicon size | Annealing Temp. |
| --- | --- | --- | --- | --- | --- |
| GAPDH | 5' ATG GTG AAG GTC GGT GTG 3' | 5' GAA CTT GCC GTG GGT AGA 3' | [NR_197270.1](https://www.ncbi.nlm.nih.gov/entrez/viewer.fcgi?db=nucleotide&id=2778098898) | 162bp | 56 |
| Bax | 5' CGG CGA ATT GGA GAT GAA CTG G 3' | 5' CAT GCA AAG TAG AGG GCA ACC 3' | [NM_017059.2](https://www.ncbi.nlm.nih.gov/entrez/viewer.fcgi?db=nucleotide&id=386869282) | 164bp | 58.4 |
| Bcl-2 | 5' TGT GGA TGA CTG ACT ACC TGA ACC 3' | 5' CAG CCA GGAGAA ATC AAA CAG AGG 3' | [NM_016993.2](https://www.ncbi.nlm.nih.gov/entrez/viewer.fcgi?db=nucleotide&id=1937369785) | 122bp | 58.1 |
| VEGF-A | 5' GCA ATG ATG AAG CCC TGG AC 3' | 5' GGT GAG GTT TGA TCC GCA TG 3' | [NM_001287114.1](https://www.ncbi.nlm.nih.gov/entrez/viewer.fcgi?db=nucleotide&id=560186593) | 78bp | 58 |
| 5α-reductase | 5' GAC CAC AGG CGA GAT GCA GA 3' | 5' TGT GTT TCC CGT AAC TGG CG 3' | [NM_022711.5](https://www.ncbi.nlm.nih.gov/entrez/viewer.fcgi?db=nucleotide&id=1938523151) | 122bp | 62 |
| Androgene Receptor | 5' CTC TCT CAA GAG TTT GGA TGG CT 3' | 5' CAC TTG CAC AGA GAT GAT CTC TGG 3' | [NM_012502.2](https://www.ncbi.nlm.nih.gov/entrez/viewer.fcgi?db=nucleotide&id=1937370033) | 342bp | 61 |

Supplementary Table 1: Primers Sequences
